# Laboratory evolution of *Mycobacterium* on agar plates for analysis of resistance acquisition and drug sensitivity profiles

**DOI:** 10.1038/s41598-021-94645-z

**Published:** 2021-07-23

**Authors:** Tomoya Maeda, Masako Kawada, Natsue Sakata, Hazuki Kotani, Chikara Furusawa

**Affiliations:** 1 RIKEN Center for Biosystems Dynamics Research, 6-2-3 Furuedai, Suita, Osaka 565-0874 Japan; 2 Universal Biology Institute, The University of Tokyo, 7-3-1 Hongo, Tokyo, 113-0033 Japan; 3 Present Address: Laboratory of Microbial Physiology, Research Faculty of Agriculture, Hokkaido University, Kita 9, Nishi 9, Kita-ku, Sapporo, Hokkaido 060-8589 Japan

**Keywords:** Evolution, Genetics, Microbiology

## Abstract

Drug-resistant tuberculosis (TB) is a growing public health problem. There is an urgent need for information regarding cross-resistance and collateral sensitivity relationships among drugs and the genetic determinants of anti-TB drug resistance for developing strategies to suppress the emergence of drug-resistant pathogens. To identify mutations that confer resistance to anti-TB drugs in *Mycobacterium* species, we performed the laboratory evolution of nonpathogenic *Mycobacterium smegmatis*, which is closely related to *Mycobacterium tuberculosis*, against ten anti-TB drugs. Next, we performed whole-genome sequencing and quantified the resistance profiles of each drug-resistant strain against 24 drugs. We identified the genes with novel meropenem (MP) and linezolid (LZD) resistance-conferring mutation, which also have orthologs, in *M. tuberculosis* H37Rv. Among the 240 possible drug combinations, we identified 24 pairs that confer cross-resistance and 18 pairs that confer collateral sensitivity. The acquisition of bedaquiline or linezolid resistance resulted in collateral sensitivity to several drugs, while the acquisition of MP resistance led to multidrug resistance. The MP-evolved strains showed cross-resistance to rifampicin and clarithromycin owing to the acquisition of a mutation in the intergenic region of the Rv2864c ortholog, which encodes a penicillin-binding protein, at an early stage. These results provide a new insight to tackle drug-resistant TB.

## Introduction

Tuberculosis (TB) is one of the top ten causes of death, exceeding HIV as the leading cause of death from a single infectious agent^1^. In 2018, 1.5 million TB-related deaths were reported worldwide^1^. Although the global rate of TB incidence is decreasing at approximately 2% per year^1^, the rapid emergence of multidrug-resistant TB strains poses a serious threat to global public health. Therefore, the coverage and quality of diagnosis, treatment, and care for individuals with multidrug-resistant TB should be improved.

The molecular diagnosis of drug-resistant *Mycobacterium tuberculosis* relies on genotypic diagnostics as well as on drug susceptibility testing^2^. Although mutations that confer resistance to several anti-TB drugs have been identified by whole-genome sequencing of clinical isolates of *M. tuberculosis*^3–7^, it is difficult to detect mutations that confer resistance to relatively novel or minor drugs, whose potential resistance is not evaluated. Many second-line drugs included in treatment regimens for drug-resistant TB infections, such as d-cycloserine, are highly toxic^8^. Therefore, to minimize mortality related to multidrug-resistant TB infection, it is important to treat patients using anti-TB drugs in a minimal toxicity regimen that is capable of eliminating the infecting strains. However, the current molecular diagnostic strategies for drug-resistant TB are limited owing to the incomplete identification of resistance-conferring mutations and data on anti-TB drug susceptibility profile for each strain harboring the mutation.

Since the development of novel drugs requires a large upfront investment and long-term clinical trials, alternative approaches to combat antimicrobial resistance (AMR) are also required urgently. One possible strategy to combat AMR is the application of evolutionary trade-offs to suppress further resistance since the emergence of AMR is based on evolutionary dynamics^9–12^. The acquisition of antibiotic resistance towards the first drug may potentially lead to a concomitant increase or decrease in susceptibility to other drugs, which are known as collateral sensitivity or cross-resistance, respectively^13^. Cross-resistance can be observed among the different classes of antibiotics as well as all the antibiotics belonging to the same class due to a single mechanism e.g. enhancement of drug efflux and inhibition of drug uptake^14–17^. Therefore, understanding such cross-resistance interactions is important for determining the drug combinations for treatments. Antibiotic pairs that exhibit collateral sensitivities and the corresponding genetic determinants have been studied extensively through parallel laboratory evolution in whole-genome sequencing and phenotyping assays^14,16–20^. These studies identified novel collateral-sensitivity and cross-resistance interactions and proposed new, rational treatment strategies to exploit collateral sensitivity^9,17^. However, such cross-resistance and collateral-sensitivity interactions in *M. tuberculosis* have not been investigated comprehensibly thus far.

In this study, to identify the mutations that confer resistance to anti-TB drugs and the novel collateral-sensitivity and cross-resistance interactions, we performed the laboratory evolution of non-pathogenic *M. smegmatis* under treatment with ten anti-TB drugs. *M. smegmatis* is commonly used in laboratory experiments on the *Mycobacterium* genus. We show that thioridazine (TRZ) is the ideal drug among the ten drugs for suppressing resistance acquisition. For each obtained evolved strain, we performed whole-genome sequencing and phenotyping assays to evaluate the process by which the acquisition of resistance to one drug changes resistance and susceptibility to the 24 other drugs. These results helped identify novel resistance-conferring mutations as well as the patterns of cross-resistance and collateral sensitivity. We also showed that the acquisition of Meropenem (MP) resistance owing to the newly identified mutation in the gene encoding penicillin-binding protein (PBP) confers multidrug-resistance to the strain. This is the first study to report the comprehensive laboratory evolution of *Mycobacterium* with respect to anti-TB drug resistance.

## Results

### Laboratory evolution of *M. smegmatis* under treatment with ten anti-TB drugs

**Figure 1:**
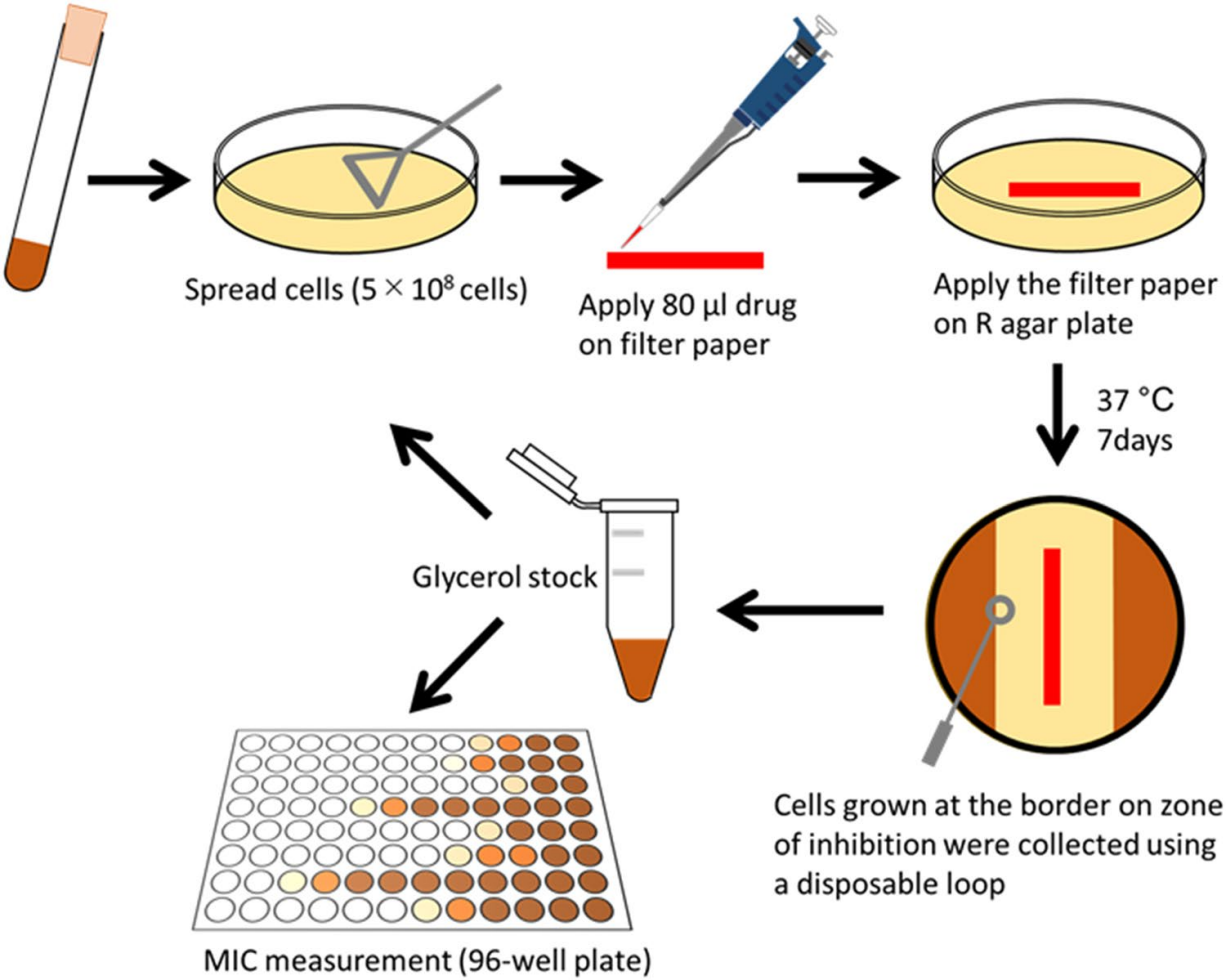
Schematic overview of laboratory evolution. In this experiment, 5 × 10^8^ cells were spread on an R agar plate, and a filter paper soaked with the corresponding drug solution was placed on the surface of the culture. The plate was incubated for 7 days at 37 °C. After incubation, the colonies at the border of the zone of inhibition were collected on an inoculation loop, and 5 × 10^8^ cells were deposited on a fresh R agar plate, and a drug-soaked filter paper was placed following the same method as that followed on the first day of the experiments. The cells transferred at each passage were stocked in glycerol at − 80 °C for minimum inhibitory concentrations (MIC) measurements.

For laboratory evolution, we selected three first-line anti-TB drugs, i.e. isoniazid (INZ), rifampicin (RIF), and ethambutol (EMB); five second-line anti-TB drugs, i.e. d-cycloserine (DCS), bedaquiline (BDQ), linezolid (LZD), meropenem (MP), and clofazimine (CFZ); and two potential anti-TB drugs, i.e. clarithromycin (CM), and thioridazine (TRZ) (Table 1). Figure 1 shows a schematic overview of the laboratory evolution protocol. Since *M. smegmatis* forms aggregates in liquid media, we developed a new method for laboratory evolution on an agar plate under a drug gradient. Compared to those used in previous studies^16^, this laboratory evolution protocol did not require an excess number of serially diluted drug gradient cultures; therefore, the method was easy and labor-friendly. Using *M. smegmatis* NCTC8159 as a wild-type strain, the laboratory evolution experiments were repeated for three to ten passages until the populations showed at least two-fold increase in MIC, or for ten passages. To evaluate the reproducibility of evolutionary dynamics, independent culture lines (three for the first-line anti-TB drugs (i.e. INZ, RIF, and EMB) and four for the other drugs) were propagated in parallel.

**Table 1:** List of anti-tuberculosis drugs used in this study.

Drug	Abbreviation	Category	Biological target
**Used for laboratory evolution**			
Bedaquiline	BDQ	Bedaquiline	ATP synthase
Clofazimine	CFZ	Clofazimine	DNA
Clarithromycin	CM	Macrolide	Ribosome
d-Cycloserine	DCS	Cycloserine	Peptidoglycan layer
Ethambutol	EMB	Ethambutol	Fatty acid biosynthesis
Isoniazid	INZ	Hydrazide	Fatty acid biosynthesis
Linezolid	LZD	Oxazolidinone	Ribosome
trihydrate	MP	Carbapenem	Peptidoglycan layer
Rifampicin	RIF	Rifamycin	RNA polymerase
Thioridazine	TRZ	Phenothiazine	Ion channel transport
**Only used for drug susceptibility measurements**			
Amikacin	AMK	Aminoglycoside	Ribosome
Arginine	ARG	Amino acid	Others
Capreomycin	CPM	Polypeptide	Ribosome
Chloramphenicol	CP	Chloramphenicol	Ribosome
Ciprofloxacin	CPFX	Fluoroquinolone	DNA
Erythromycin	EM	Macrolide	Ribosome
Trimethoprim	TMP	Antifolate	Folate biosynthesis
Ethionamide	ENM	Hydrazide	Fatty acid biosynthesis
Phleomycin	PLM	Bleomycin	DNA
Rifabutin	RFB	Rifamycin	RNA polymerase
Rifamycin SV	RFM	Rifamycin	RNA polymerase
Streptomycin	SM	Aminoglycoside	Ribosome
Tetracycline	TET	Tetracycline	Ribosome
Thiocarbohydrazide	TCH	Hydrazide	Others

**Figure 2:**
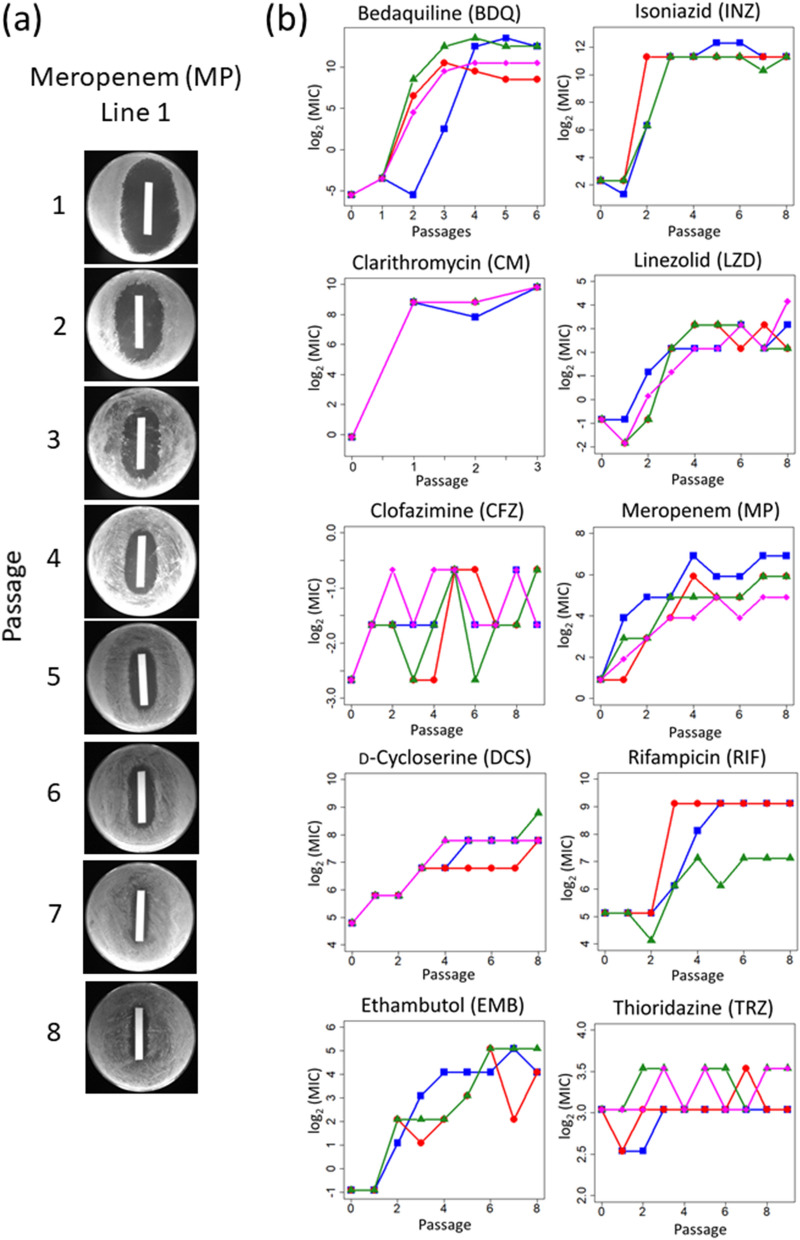
Resistance time series for laboratory evolution. (**a**) Representative images of the culture plates from the laboratory evolution experiment. The images of MP-evolved line 1 are shown. The filter papers were soaked in 10 mg ml^−1^ MP. (**b**) Time series of minimum inhibitory concentrations (MIC) calculation during the laboratory evolution experiment. The MIC values (log_2_(MIC [μg ml^−1^]) of three or four independent culture lines (line 1: blue, line 2: red, line 3: green, and line 4: magenta) are plotted.

Figure 2a shows an agar plate at each passage during laboratory evolution. The zone of inhibition on the agar plates gradually decreased with each passage (Table S1). After laboratory evolution, the MIC values for the evolved population at each passage were determined by the growth of the cells from the frozen glycerol stock in 96-well microplates containing the serially diluted drugs. Figure 2b shows the time course of MIC change during laboratory evolution. After the laboratory evolution experiment, we isolated a single clone from an R agar plate without drugs from the endpoint culture. We used the isolated single clones as the evolved strains for further analysis. The rapid emergence of resistance to INZ and CM was observed (after approximately two passages), while significant TRZ resistance was not observed even after nine passages, corresponding to cultivation for 70 days (Fig. 2b).

### Identification of novel mutations conferring resistance against anti-TB drugs

To identify mutations that confer resistance against anti-TB drugs, we performed genome resequencing analysis of the 37 evolved strains (Table S2). Less than 19 mutations were fixed in each of the evolved strains. We found several genes and gene functions to which mutations were commonly fixed in the evolved strains (Table 2), which suggested the contributions of these mutations to drug resistance. The strains evolved against the three first-line anti-TB drugs commonly harbored mutations associated with high-confidence drug resistance in *M. tuberculosis*, which comprised common mutations in *inhA* and *katG* in INZ-evolved strains, *rpoB* in RIF-evolved strains, and *embABC* in EMB evolved strains (Table 2). These results indicate that the laboratory evolution protocol faithfully replicated the evolutionary dynamics in clinical isolates that were resistant to these drugs. All four BDQ-evolved strains had mutations in *atpE*, which encodes the ATP synthase F_O_ subunit C, which had been previously identified in both in vitro-selected and clinically isolated resistant *M. tuberculosis* strains (Table 2).

**Table 2:** Representative mutations that were commonly fixed in the evolved strains.

Anti-TB drugs	Effect	Locus tag	Ortholog in MTB*	Description	Previously reported**
BDQ	Non-synonymous	AT701_RS24130	*atpE*	ATP synthase Fo subunit C	Y
CFZ	Promoter	AT701_RS31085/AT701_RS31090		Metallophosphoesterase/hypothetical protein	
CMZ					
DCS	Non-synonymous	AT701_RS01985		EamA family transporter	
	Non-synonymous	AT701_RS01990		PLP-dependent aminotransferase family protein	
EMB	Non-synonymous	AT701_RS31290	*embC*	Arabinosyltransferase C	Y
	Promoter	AT701_RS31295	*embA*	Arabinosyltransferase A	Y
	Non-synonymous	AT701_RS3130	*embB*	Arabinosyltransferase B	Y
INZ	Non-synonymous	AT701_RS15800	*inhA*	NADH-dependent enoyl-ACP reductase	Y
	Frame-shift	AT701_RS3127	*katG*	Catalase-peroxidase	Y
LZD	Frame-shift	AT701_RS07195	*oxiR*	TetR/AcrR family transcriptional regulator	N
MP	Promoter	AT701_RS30910	Rv2864c	Penicillin-binding protein	N
	Non-synonymous	AT701_RS30910	Rv2864c	Penicillin-binding protein	N
	Frame-shift	AT701_RS31385	Rv3811	Putative *N*-acetylmuramoyl-l-alanine amidase	N
TRZ	Non-synonymous	AT701_RS13855	Rv2707	YihY/virulence factor BrkB family protein	N
RIF	Non-synonymous	AT701_RS07130	*rpoB*	DNA-directed RNA polymerase subunit beta	Y

*Orthlog genes in *M. tuberculosis* H37Rv strain are shown.

**Genomic mutations of *M. tuberculosis* associated with drug resistance are indicated as Y (others are indicated as N) based on the previous report (Lange et al.^7^).

We also identified commonly fixed mutations in anti-TB drug-evolved strains. MP is a β-lactam antibiotic from the carbapenem group that has been recommended for use in longer multidrug-resistant TB regimens by WHO after 2018^21^. To the best of our knowledge, MP resistance-conferring mutations have not been reported thus far in clinical isolates of *M. tuberculosis*. In this study, three out of the four MP-evolved strains harbored three mutations in the IGR between AT071_RS30905, which encodes an SGNH/GDSL hydrolase family protein, and AT701_RS30910, which encodes a PBP (ortholog of Rv2864c in *M. tuberculosis* H37Rv); the coding region of AT701_RS30910; and the coding region of AT701_RS31385, which encodes a putative *N*-acetylmuramoyl-l-alanine amidase (ortholog of Rv3811 in *M. tuberculosis* H37Rv) (Table 2). Since MP targets PBPs, and MP resistance-conferring mutations have been identified in genes encoding PBPs in clinical isolates of other pathogens, such as *Acinetobacter baumannii*^22,23^, these commonly fixed mutations probably contribute to the acquisition of MP resistance. Mutations in genes encoding transporters i.e. AT701_RS28865 (ortholog of Rv3501c in *M. tuberculosis* H37Rv strain) and *sugA* were also commonly found in MPE2 and MPE3 strains. Besides, a mutation in *lpqY* encoding an ABC transporter was also identified in MPE1 strains. These transporters may be involved in MP resistance.

The four LZD-evolved strains harbored mutations in the AT701_RS07195, which encodes the TetR/AcrR family transcriptional regulator (ortholog of *oxiR* in *M. tuberculosis* H37Rv strain) (Table 2). LZDE2 (the LZD-evolved strain derived from line 2) contained a mutation in the IGR, while the other three strains had mutations in the coding region. It was reported in a recent study that the overexpression of *oxiR* resulted in increased INZ resistance in *M. tuberculosis*^24^. Although the previous study did not examine the contribution of *oxiR* to LZD resistance, these results suggest that regulation by *oxiR* affects susceptibility to several anti-TB drugs, including LZD and INZ. Previous studies have shown that mutations in *rrl*, which encodes 23S rRNA, confer LZD resistance in several pathogens, including *M. tuberculosis*, *Staphylococcus* spp., and *Enterococcus* spp.^25–27^. Since *M. smegmatis* NCTC8159 carries two copies of *rrl* genes, our short-read sequencing analysis may fail to map mutations in the *rrl* genes. Therefore, we performed Sanger sequencing analyses using the specific primer sets. However, none of the LZD evolved strains harbored a mutation in *rrl* was detected.

We also identified commonly fixed mutations in the evolved strain that have not been reported in *M. tuberculosis*. Three out of the four CFZ-evolved strains (CFZE1, CFZE2, and CFZE4) and DCS-evolved strains (DCSE2, DCSE3, and DCSE4) commonly contained mutations in the IGR between AT701_RS31085, which encodes a metallophosphoesterase, and AT701_RS31090, which encodes a protein of unknown function, and in AT701_RS01990, which encodes a PLP-dependent aminotransferase family protein respectively (Table 2). Additionally, both DCS3 and DCSE4 strains harbored mutations in AT701_RS01985, which encodes the EamA family transporter (Table 2). The *M. tuberculosis* H37Rv strain did not have the orthologs of these genes suggesting the existence of specific mechanisms for the acquisition of CFZ and DCS resistances by *M. smegmatis*. The DCSE1 strain harbored a mutation in the IGR of AT701_RS12100 (ortholog of *ddlA* in *M. tuberculosis* H37Rv strain), which encodes a d-alanine-d-alanine ligase (Table S2). A previous study also showed that the overexpression of *ddlA* conferred DCS resistance in *M. smegmatis*^28^. In contrast, the mutations that confer CM and TRZ resistances remain unclear. We did not identify any common mutations in the CM-evolved strains. Although two TRZ-subcultured strains (TRZE2 and TRZE3) harbored an L45Y mutation in AT701_RS13855 (ortholog of Rv2797 in *M. tuberculosis* H37Rv), which encodes the BrkB family YihY virulence factor, these strains did not exhibit increased TRZ resistance. Therefore, the contribution of the AT701_RS13855 mutation to TRZ resistance is unclear.

### Quantification of cross-resistance and collateral sensitivity

To investigate the mechanism by which the acquisition of resistance to one drug changes the overall drug resistance profile in each strain, we measured the MICs of the 24 drugs shown in Table 1 for each evolved strain (912 measurements × 3 replicates) relative to the parent strain (Table S3). Cross-resistance was observed, where 24 pairs of drugs exhibited significant cross-resistance within the possible 240 combinations (Mann–Whitney U-test, FDR < 0.05, and mean relative MIC > 2^0.5^ fold). Conversely, 18 pairs of drugs exhibited collateral sensitivity (Mann–Whitney U-test, FDR < 0.05, and mean relative MIC < 2^–0.5^ fold).

**Figure 3:**
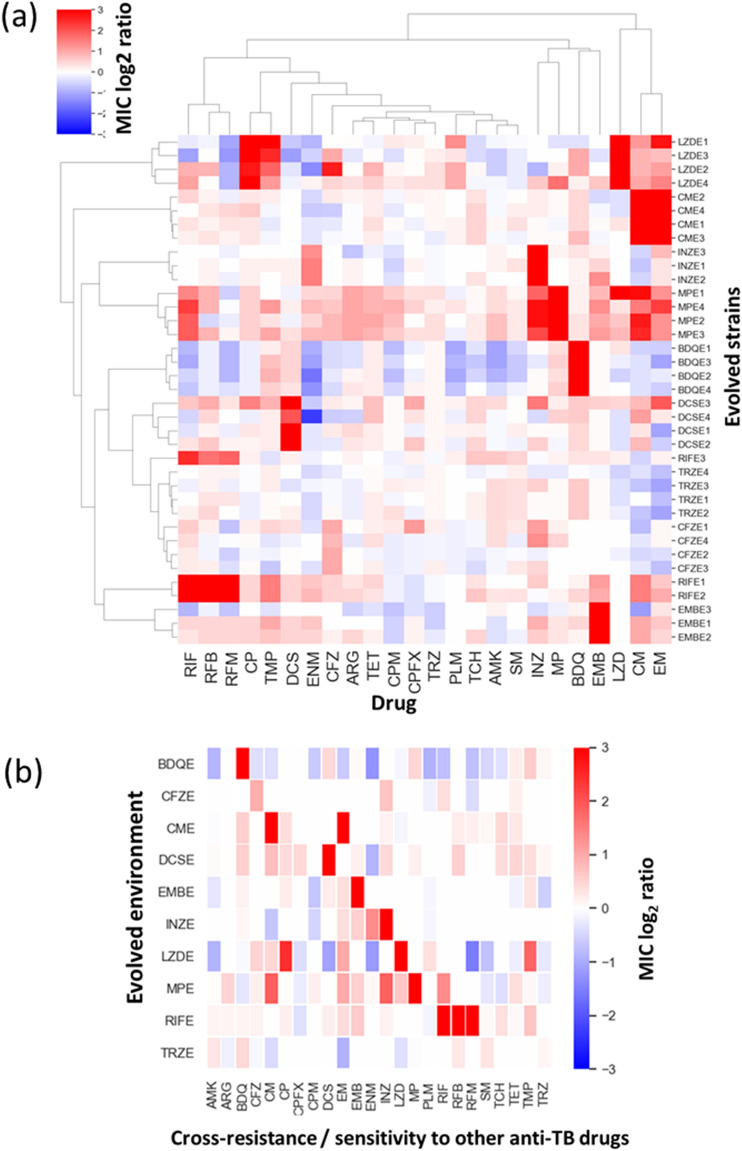
Consequence of anti-tuberculosis (TB) drug resistance evolution: cross-resis and collateral sensitivity. (**a**) Heatmap representing the quantification of anti-TB drug susceptibilities in the 37 evolved strains. The color coding represents the increase (red) or decrease (blue) in minimum inhibitory concentrations (MICs) relative to that in the wild-type (NCTC8159) strain. For each strain, the MICs for three replicates of the evolved strains and twenty replicates of the wild-type strains were calculated. The order of anti-TB drugs and evolved strains was determined by hierarchical clustering using the function seaborn.clustermap in Seaborn library of Python 3.7. (**b**) The identified combinations of drugs that exhibited either cross-resistance or collateral sensitivity for each evolved strain in the same environment. The combinations were determined using the Mann–Whitney U test (FDR < 0.05), and the colors indicate resistance to the stress relative to the parent strain. The heatmap was generated using the function seaborn.heatmap in Seaborn library of Python 3.7.

As shown in the hierarchical clustering (Fig. 3a), the drug resistance profiles were generally similar among evolved strains derived using the same selection pressure, with few exceptions (LZDE3 and EMBE3 strains). These results suggest that independently evolved strains acquired a similar phenotype. The RIF-, CM-, and INZ-evolved strains showed cross-resistances to drugs from the same family, i.e., to other drugs of the rifamycin family, such as rifabutin (RFB) and rifamycin SV (RFM), in RIF-evolved strains; to another hydrazide drug ethionamide (ENM) in INZ-evolved strains; and to another macrolide drug erythromycin (EM) in CM-evolved strains (Fig. 3b). Cross-resistance was also observed among drugs with different mechanisms of action. For example, all four MP-evolved strains exhibited cross-resistances to the three first-line anti-TB drugs, macrolide drugs, and arginine (ARG) (Fig. 3b). These results indicate that the acquisition of MP resistance causes multidrug resistance. The four LZD-evolved strains also acquired a multidrug-resistant phenotype; however, the number of cross-resistant drugs was lesser than the number of MP-evolved strains. The LZD-evolved strains commonly exhibited cross-resistance to chloramphenicol (CP) and trimethoprim (TMP) (Table S3). Since the four LZD-evolved strains commonly harbored mutations in the *oxiR* ortholog, this mutation may confer cross-resistance to CP and TMP. The DCSE3 strain exhibited cross-resistances to 12 out of the 24 drugs, although the three other DCS-evolved strains commonly exhibited cross-resistance to two drugs (BDQ and CM). Since only the DCSE3 strain harbored a mutation in AT701_RS17110, which encodes an MFS transporter (ortholog of Rv1200 in *M. tuberculosis* H37Rv), the MFS transporter might play a role in multidrug efflux. These results suggest the existence of an alternative evolutionary pathway for the acquisition of DCS resistance along with multidrug resistance.

Along with a novel cross-resistance relationship, we also identified novel collateral sensitivity relationships among drugs. Among the ten anti-TB drugs used for the laboratory evolution experiments, the MICs of multiple drugs were lowered for BDQ- and LZD-evolved strains. BDQ evolved strains tended to decrease in MICs of two rifamycin family drugs (RIF and rifamycin SV (RFM)), two aminoglycoside drugs (streptomycin (SM) and amikacin (AMK)), and two macrolide family drugs (EM and CM) (Fig. 3). Reduced CFU of BDQ evolved strains compared to that of the parent strain in the presence of AMK and CM was also confirmed by CFU assay (Fig S2). These results suggest that these drugs can efficiently kill BDQ-resistant *Mycobacteria*. The MIC of CM was reduced by more than 1.4-fold for the three INZ-evolved strains (Fig. 3). The LZD-evolved strains tended to exhibit collateral sensitivities to ENM and DCS (Fig. 3). Compared to the other evolved strains derived from the same selection pressure, the LZDE3 and EMBE3 strains exhibited collateral sensitivities to a relatively large number of drugs (Fig. 3). These data suggest the existence of alternative paths for the acquisition of LZD or EMB resistance at a higher fitness cost. Interestingly, the four strains isolated from the endpoint cultures under TRZ selection pressure exhibited collateral sensitivity to EM. Also, three of the four TRZ strains showed cross-resistance to BDQ, even though these did not exhibit increased TRZ resistance (Fig. 3). These results suggest that cross-resistance and/or collateral sensitivity can be acquired before the acquisition of resistance under the given selection pressure.

### Association of collateral sensitivity with reduced growth rates

**Figure 4:**
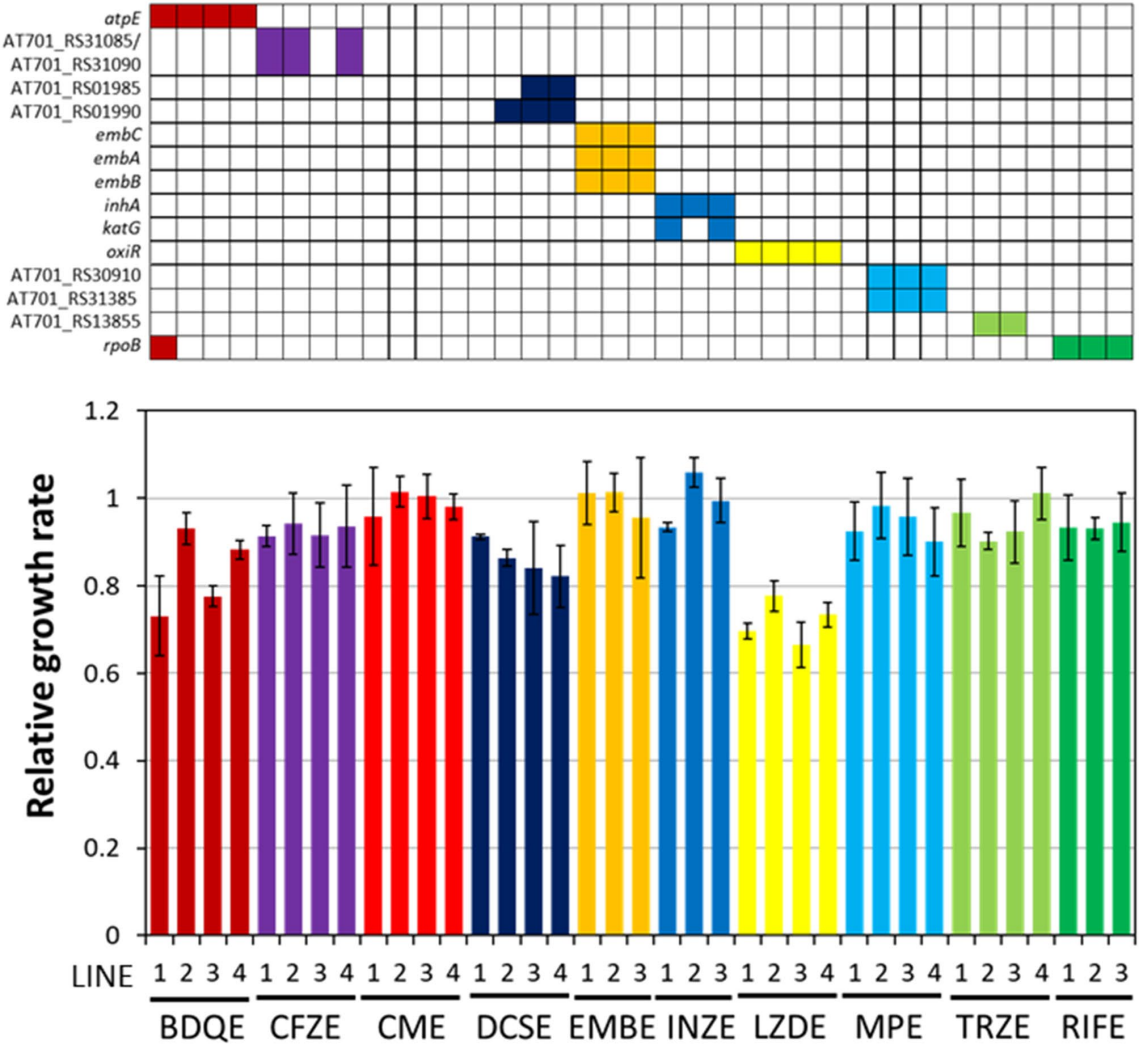
Relative growth rates of the 37 evolved strains in drug-free medium. Growth rates of the 37 evolved strains and the wild-type strain in drug-free R liquid medium were measured. Commonly identified mutations are shown in the colored boxes at the top of the figure. Each measurement was performed in triplicate, starting from independent cultures. Each column represents the relative growth rate (evolved strain/wild type strain) on the *x*-axis.

A previous study showed that antibiotic resistance generally confers a measurable fitness cost, i.e., significantly reduced growth in the antibiotic-free medium^14,29^. To investigate whether the collateral sensitivities were associated with reduced growth, we measured the growth rates of the 37 evolved strains and the parent strains in antibiotic-free media. Twenty-seven out of the thirty-seven evolved strains showed less than 10% reduction in growth rates. Among the ten anti-TB drugs used in the laboratory evolution experiment, resistance to LZD was associated with the lowest growth rate (reduced growth rates in the range of 22–33%) (Fig. 4). The LZDE3 strain showed the lowest growth rate (33%), which suggested the correlation between reduced growth rate and a large number of collateral sensitivity profiles. The BDQ-evolved strains also tended to exhibit reduced growth rates (27% in BDQE1, 22% in BDQE3, 12% in BDQE4, and 7% in BDQE2). These results suggest that a reduced growth rate in antibiotic-free media was associated with collateral sensitivity to other drugs.

### Association between early-stage resistance acquisition to MP and multidrug resistance

**Figure 5:**
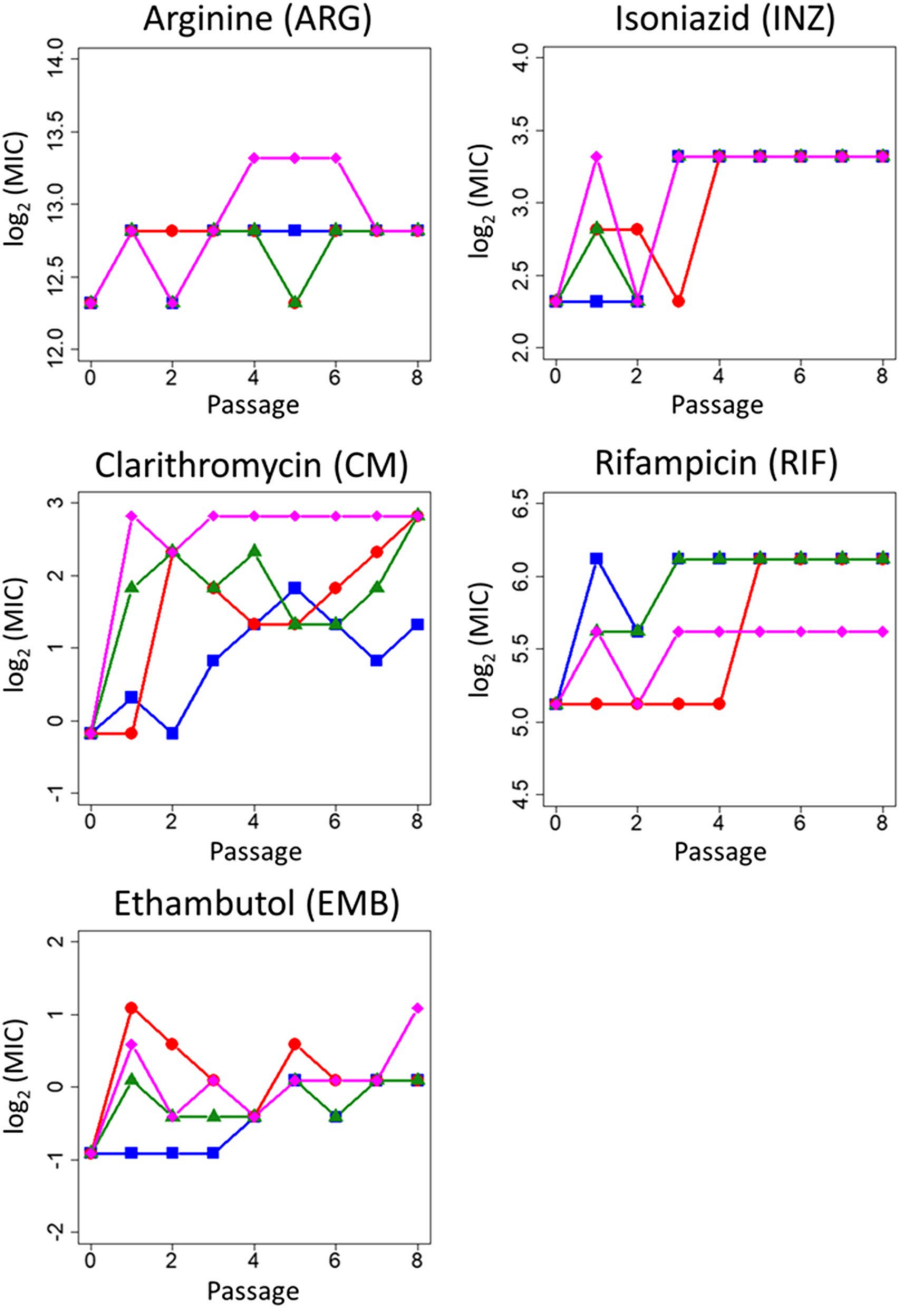
Meropenem resistance acquisition associated with multidrug resistance. The minimum inhibitory concentrations (MICs) of arginine (ARG), clarithromycin (CM), ethambutol (EMB), isoniazid (INZ), and rifampicin (RIF) for the MP-evolved populations at each passage were determined. The MIC values (log_2_(MIC [μg ml^−1^]) of four independent culture lines (line 1: blue, line 2: red, line 3: green, and line 4: magenta) compared to the wild-type strain are plotted.

Since the four MP-evolved strains commonly exhibited a multidrug resistance profile, we further evaluated the time development of multidrug resistance during MP resistance evolution. As shown in Fig. 3a, the MP-evolved strains commonly exhibited cross-resistance to INZ, EMB, RIF, CM, and ARG. We measured the MICs of these five drugs for the MP-evolved population at each passage by measuring the growth of the cells from the frozen glycerol stock (Fig. 5). Cells from the four lines exhibited cross-resistances to the five drugs within two to six passages (Fig. 5). These results indicate that multidrug resistance is acquired at an early stage of MP resistance acquisition. Next, we isolated single clones from the glycerol stock of each line at passage two or three, the MICs for which were comparable to those of the corresponding population. Based on findings from the Sanger sequencing experiment, we observed that among the three commonly fixed mutations in MP-evolved strains (Table 2), these clones carried mutations only in the IGR between AT071_RS30905 and AT701_RS30910 consistently. The three isolated clones commonly exhibited cross-resistances to RIF and CM as well as a 16-fold increase in MP resistance (Fig. S1). These results strongly suggest that the mutations in the IGR confer multidrug resistance along with MP resistance.

## Discussion

In this study, we performed the laboratory evolution of a nonpathogenic model *Mycobacteria* strain under treatment with anti-TB drugs. We also investigated the genotypic and phenotypic changes in the evolved strains. From these experiments, novel information was obtained about mutations that lead to anti-TB drug resistance and alterations in the susceptibility patterns to anti-TB drugs in the evolved strains *M. smegmatis* is a non-pathogenic and relatively fast-growing bacterium, and this species shares the same cell wall structure and 2507 orthologous clusters, including 12 out of 19 virulence genes, with *M. tuberculosis*^30,31^. Therefore, our data can be useful for understanding the basis of the evolutionary dynamics of anti-TB drug resistance in *Mycobacterium* species including *M. tuberculosis*. Our laboratory evolution protocol did not require multiple serially diluted drug gradient cultures; therefore, the method is simple and labor-friendly. A recent study using another well-known pathogenic bacterium *Pseudomonas aeruginosa* also performed laboratory evolution against antibiotics on agar plates^32^ indicating the usability of agar-based selection for laboratory evolution against antibiotics.

We identified 24 and 18 new pairs of drugs that conferred cross-resistance and collateral sensitivity, respectively (Fig. 3b). Most of these newly identified drug relationships differ from those identified in well-studied bacteria, such as *Escherichia coli* and *Pseudomonas aeruginosa*^14,16,17^. In *E. coli* and *P. aeruginosa*, the primary multidrug resistance mechanisms consist of drug transport systems, such as the multidrug efflux pump and outer membrane porin^14,16,17^. Conversely, mutations in genes that encode components of drug transport systems were not commonly identified in this study. These differences could affect the different drug sensitivity profiles between *Mycobacterium* and other pathogenic bacteria. Interestingly, while horizontal gene transfer (HGT) is a common mechanism in the acquisition of drug resistance in several bacteria, *M. tuberculosis* does not appear to participate in extensive HGT^33^. These results indicate the necessity of a thorough investigation of the difference between drug-resistance mechanisms in different pathogenic bacteria for tackling drug resistance.

We identified genes with novel MP resistance-conferring mutations, the orthologs of which are also present in *M. tuberculosis* H37Rv (i.e., orthologs of Rv2864c and Rv3811) (Table 2). The mutations could confer MP resistance in *M. tuberculosis*; therefore, it is necessary to confirm whether these mutations are found in clinical isolates of MP-resistant *M. tuberculosis* strains. Clinical isolates of MP-resistant *M. tuberculosis* strains have not been reported to date^7^, possibly owing to the short history of the clinical use of MP (WHO recommendation after December 2018). Although carbapenem antibiotics are considered the “last resort” drugs for a variety of bacteria, clinical isolates of MP-resistant *M. tuberculosis* strains may emerge shortly. Since our laboratory evolution experiment demonstrated that the acquisition of MP resistance at an early stage led to the acquisition of multidrug resistance (Fig. 3), the clinical use of MP may be a potential risk factor for the alteration of the multidrug resistance status in *M. tuberculosis*. The mutations in both the IGR and open reading frame (ORF) of AT701_RS30910 encoding a PBP were commonly identified in the MP-evolved strains (Table 2). Mutations in the genes encoding PBPs were also identified as carbapenem-resistance conferring mutations in various bacteria. In *A. baumannii*, the A517V mutation in *ftsI* (PBP3) conferred four-fold increased MP resistance owing to a reduction in drug-binding affinity^23^. It was also reported that mutations in *ftsI* as well as in *mrdA*, which encodes another PBP, contribute to carbapenem resistance in *E. coli*^34^. Although AT701_RS30910 is not an ortholog of *ftsI* or *mrdA*, meropenem may bind strongly to the PBP encoded by AT701_RS30910 compared to other PBPs in *M. smegmatis*. The acquisition of IGR mutations in AT701_RS30910 led to 16-fold increase in MP resistance, while the MP-evolved strains showed 32-fold increase in MP resistance. These results indicate that while MP resistance is primarily acquired through IGR mutation, the other two mutations (i.e., mutations in the ORF of AT701_RS30910 and the ORF of AT701_RS31385, which encodes a putative *N*-acetylmuramoyl-l-alanine amidase) also confer MP resistance. Since the IGR mutation was first acquired in the three independent MP-evolved lines, the order of mutation acquisition leading to MP resistance may be important. Since the IGR mutation also confers RIF and CM resistance (Fig. S1), this mutation may affect the expression of other genes related to RIF and CM resistance. It was reported that mutations in the DivIVA homolog Wag31 which plays important role in peptidoglycan synthesis, cell growth, and cell division, maintained resistance to lipophilic drugs (RIF, EM, CFZ, and novobiocin)^35^. Similar to the result of mutations in *wag31*, the alteration of peptidoglycan synthesis activity in response to IGR mutation in AT701_RS30910 may also affect the resistance to lipophilic drugs. To verify the effects of the common mutations found in the evolved strains, introductions of such mutations to the parent strain is necessary. However, our attempts to construct the reconstructed mutant strains using the NCTC8159 strain failed. Therefore, further validations remain as future works.

TRZ resistance was not observed in any strain during the laboratory evolution under TRZ selection pressure. Although populations of the two independent TRZ-evolved lines at the endpoint of the laboratory evolution showed 1.4-fold increased TRZ resistance (Fig. 2b), we failed to isolate single clones showing increased TRZ resistance. This different resistance between the population and the isolated single clones could be due to heterogeneity in the population level. In addition, cross-resistance to TRZ was not above 2^0.5^-fold in any of the evolved strains. TRZ, an antipsychotic drug that is a phenothiazine derivative, has been shown to exhibit antimycobacterial properties both in vitro and in vivo^36–38^. To the best of our knowledge, neither TRZ-resistant *M. tuberculosis* strains nor mutations conferring resistance to TRZ have been reported to date. TRZ exhibited considerable efficacy in the treatment of XDR-TB owing to the multi-mechanisms of action, including efflux pump-inhibiting activity, alteration of cell-envelope permeability, and inhibition of type-II NADH-menaquinone oxidoreductase (NDH-2)^38–40^. Although further extensive study is required to validate, it may be difficult for *Mycobacterium* strains to acquire TRZ resistance*.*

## Methods

### Bacterial strains and culture conditions

*Mycobacterium smegmatis* NCTC8159 (JCM6386), which was obtained from the Microbe Division in RIKEN-BRC (BioResource Research Center, Tsukuba-shi, Japan), was used as the parent strain. *M. smegmatis* cells were cultured in R medium (a medium designated for this strain by RIKEN JCM) containing 10.0 g l^−1^ bacto peptone (BD-Difco, Franklin Lakes, New Jersey), 5.0 g l^−1^ yeast extract (BD-Difco), 5.0 g l^−1^ malt extract (BD-Difco), 5.0 g l^−1^ casamino acids (BD-Difco), 2.0 g l^−1^ beef extract (BD-Difco), 2.0 g l^−1^ glycerol, 50.0 mgl^−1^ Tween 80, and 1.0 g l^−1^ MgSO_4_·7H_2_O (pH 7.2).

### Laboratory evolution

Before laboratory evolution, *M. smegmatis* NCTC8159 strain was cultivated in R medium without drugs used for laboratory evolution for approximately 50 generations to acclimatize them to the R medium. After 50 generation cultivation, a glycerol stock of the cells was stored as the parent strain at – 80 °C for further experiments. The chemicals used in this study are listed in Table 1. The parent strain was cultivated in liquid R medium for 48 h at 37 °C, following which 5 × 10^8^ cells were deposited onto an R agar plate (φ 90 mm). Drug solutions were allowed to drop onto a filter paper that was placed on the agar plate. The plate was incubated for 7 days at 37 °C. After incubation, colonies from the border of the zone of inhibition were collected using a disposable loop, following which 5 × 10^8^ cells were plated on a fresh R agar plate, and a filter paper that absorbed the drug was placed. The cells transferred at each passage were stored in glycerol stocks at − 80 °C for further analysis. These steps were repeated until the culture showed at least a two-fold increase in the minimal inhibitory concentration (MIC) or for ten passages. We isolated a single clone from the drug-free R agar plate from the endpoint culture and confirmed that the MIC of the isolated single clone was almost identical to that of the corresponding population in the endpoint culture. The isolated clones were used for further analysis as the evolved strains.

### Measurement of MICs

To measure the MICs, serial dilutions of each drug were prepared in 96-well microplates containing 200 µl R medium per well with a 2^0.5^ fold drug gradient in 22 dilution steps. The drug gradients depended on the maximum fold changes in the MICs for the evolved strains against the corresponding drugs. To commence MIC measurement, the cells from the frozen glycerol stock were used to inoculate the medium in each well (30 μl of glycerol stock in 170 μl of medium per well) of 96-well microplates and cultivated with agitation at 750 rotations min^−1^ at 37 °C. Cell growth was monitored by measuring OD_600_ in each well using the 1420 ARVO microplate reader (PerkinElmer Inc.). The pre-cultured cells that were calculated to have initial OD_600_ values of 0.01 were used to inoculate the medium in each well of 96-well microplates and were cultivated under agitation at 750 rotations min^−1^ at 37 °C. After 48 h cultivation, the OD_600_ values of the cultures were measured. MIC was defined as the lowest concentration of a chemical that inhibited 90% growth such that OD_600_ < 0.15. Each MIC measurement experiment was performed at least thrice, starting with independent cultures. For the evolved strains showing reduced growth rates, the cultivation time was extended for another 72 h and confirmed that no growth was observed after the time point 48 h.

The cross-resistance and collateral sensitivities of stresses were investigated using the Mann–Whitney U-test. To detect collateral relationships between drugs A and B, we compared the MIC values corresponding to drug B for the three or four strains that evolved under drug A to that of the parent strain (twenty independent replicas). The p values were obtained using “wilcox.test” (correct = F, exact = T) in the stats package of R software. We further applied the Benjamini–Hochberg FDR control to these p values to achieve FDR < 5%. Hierarchical clustering was performed using the function seaborn.clustermap in the Seaborn library package of Python 3.7. Drugs that were identified as more than 2^0.5^-fold (step size of drug dilution) increased or decreased MIC values with statistical significance (Mann–Whitney U-test, false discovery rate (FDR) < 5%) in evolved strains were determined as cross-resistance or collateral sensitivity, respectively.

To validate our methodology for determining MICs, some of the cross-resistances were also confirmed by determining colony-forming units (CFUs). For this validation, we selected cross-resistance with CM and RIF for MP evolved strains, cross-resistance with CP for LZD evolved strains, collateral-sensitivity with CM for INZ and BDQ evolved strains, collateral sensitivity with DCS for LZD evolved strains, and collateral sensitivity with AMK for BDQ evolved strains. The parents strain NCTC8159 strain, the MP evolved strains (MPE1, MPE2, MPE3, and MPE4), LZD evolved strains (LZDE1, LZDE2, LZDE3, and LZDE4), INZ evolved trains (INZE1, INZE2, and INZE3), BDQ evolved strains (BDQE1, BDQE2, BDQE3, and BDQE4) cells were cultured in R medium for 48 h, and 10^−1^ to 10^−6^ dilutions were subsequently prepared. Next, 5 µl from each dilution and the undiluted culture was dropped on R agar plates with increasing amounts of antibiotics (two-fold increments). Plates were incubated at 37 °C for 48–72 h and colonies were counted to determine the frequency of bacteria growth at each antibiotic concentration. In the case of difficulty in counting colony numbers in the spotted cultures, 100 μl appropriately diluted cells were plated on R agar plates. Each measurement was performed in triplicate, starting from independent cultures (Fig. S2).

### Isolation of genomic DNA

The stock strains were used to inoculate 5 ml of R medium without drugs, which was used for conducting laboratory evolution, contained in test tubes for 48 h at 37 °C and 150 rpm using water-bath shakers (Personal-11, Taitec Co.). Subsequently, 300 µg ml^−1^ rifampicin was added, and the culture was further maintained for 3 h to inhibit the initiation of DNA replication. The cells were harvested by centrifugation at 25 °C and 10,000 rpm for 5 min, following which the pelleted cells were stored at – 80 °C before subjecting them to genomic DNA purification. Genomic DNA was isolated and purified using a DNeasy Blood & Tissue Kit (Qiagen). The harvested cells were first suspended in 90 μl of enzymatic lysis buffer and incubated at 80 °C for 1 h. After incubation, the sample was cooled to 30 °C, after which 90 μl of lysozyme solution was added (final concentration of lysozyme was 50 g l^−1^). The sample was incubated overnight at 37 °C. After incubation, DNA was purified in accordance with the manufacturer's instructions. The quantity and purity of the genomic DNA were determined by measuring the absorbance at 260 nm and calculating the ratio of absorbances at 260 and 280 nm (A_260_/_280_) using the NanoDrop ND-2000 spectrophotometer. The A_260_/_280_ values of all the samples were confirmed to be greater than 1.7. The purified genomic DNA samples were stored at − 30 °C before use.

### Whole-genome sequencing

Genome sequences were analyzed using the Illumina MiSeq System. A 150-bp paired-end library was generated according to the Illumina protocol and sequenced using the Illumina MiSeq System. In this study, 38 samples (37 evolved strains and 1 parent strain) with different barcodes were mixed and sequenced, which yielded approximately 140-fold coverage on average. The potential nucleotide differences were validated using BRESEQ version 0.28^41^ with a reference genome sequence (Accession: NZ_LN831039).

### *rrl* gene sequencing analysis

Since *M. smegmatis* NCTC8159 carries two copies of *rrl* genes, DNA fragments containing each *rrl* gene were amplified by PCR using the specific primers: 5′-CTCGCTGCCACTAGAGAATG-3′ and 5′-GGAATTCGAGACCGAGAATG-3′, or 5′-CGCGACGAACCTCGTATTATC-3′ and 5′-GCCTCGAACGCAAGCGAATG-3′. The PCR products were purified using the QIA quick PCR purification Kit (Qiagen). Sanger sequencing analyses were performed by Griner Japan.

## Supplementary Information

Supplementary Information 1.

Supplementary Information 2.

Supplementary Information 3.

Supplementary Information 4.

## Data Availability

Fastq files (raw sequencing data) for all evolved strains analyzed in this study are available in the DDBJ Sequence Read Archive under the accession number DRA009591.

## List of symbols

AMK: Amikacin
AMR: Antimicrobial resistance
ARG: Arginine
BDQ: Bedaquiline
CFZ: Clofazimine
CM: Clarithromycin
CP: Chloramphenicol
DCS: d-Cycloserine
EMB: Ethambutol
HGT: Horizontal gene transfer
INZ: Isoniazid
LZD: Linezolid
MIC: Minimal inhibitory concentration
MP: Meropenem
ORF: Open-reading frame
PBP: Penicillin-binding protein
RIF: Rifampicin
SM: Streptomycin
TB: Tuberculosis
TMP: Trimethoprim
TRZ: Thioridazine
