# Supplementary material for: Laboratory evolution of Mycobacterium on agar plates for analysis of resistance acquisition and drug sensitivity profiles
Source: Sci Rep. 2021 Jul 23;11:15136. doi: 10.1038/s41598-021-94645-z (PMC8302736; doi:10.1038/s41598-021-94645-z)
Supplement: Supplementary file 1 — Supplementary Information 1. [file 41598_2021_94645_MOESM1_ESM.pdf]

Supplementary Materials for

Laboratory evolution of *Mycobacterium* on agar plates for analysis of resistance acquisition and drug sensitivity profiles

Tomoya Maeda[1#], Masako Kawada[1], Natsue Sakata[1], Hazuki Kotani[1], Chikara Furusawa[1,2]

[1]RIKEN Center for Biosystems Dynamics Research, 6-2-3 Furuedai, Suita, Osaka 565-0874, Japan

[2]Universal Biology Institute, The University of Tokyo, 7-3-1 Hongo, Tokyo 113-0033, Japan

#Address correspondence to Tomoya Maeda, tomoya.maeda@chem.agr.hokudai.ac.jp

&Current address: tomoya.maeda@chem.agr.hokudai.ac.jp, Laboratory of Microbial Physiology, Research Faculty of Agriculture, Hokkaido University, Kita 9, Nishi 9, Kita-ku, Sapporo, Hokkaido, 060-8589, Japan

Running title: Laboratory evolution of *Mycobacterium* on agar plates

Table S1. Drug concentrations and zones of inhibition during the laboratory evolution.

The drug concentration (μg/ml) at each passage used for the laboratory evolution is shown (a). The zone of inhibition (mm) at each passage is shown (b).

Table S2. Related to Table 2. All mutations identified in the evolved strains.

Table S3. Related to Fig. 3. Minimum inhibitory concentrations (MICs) of the 24 drugs for each evolved strain.

The MIC values (log_2(MIC [μg/ml]) for three replicates of the evolved strains and twenty replicates of the wild-type strains are shown (a). The mean relative MIC values (log_2 ratio) of the 24 drugs for 37 evolved strains compared to the wild-type strain are shown (b).

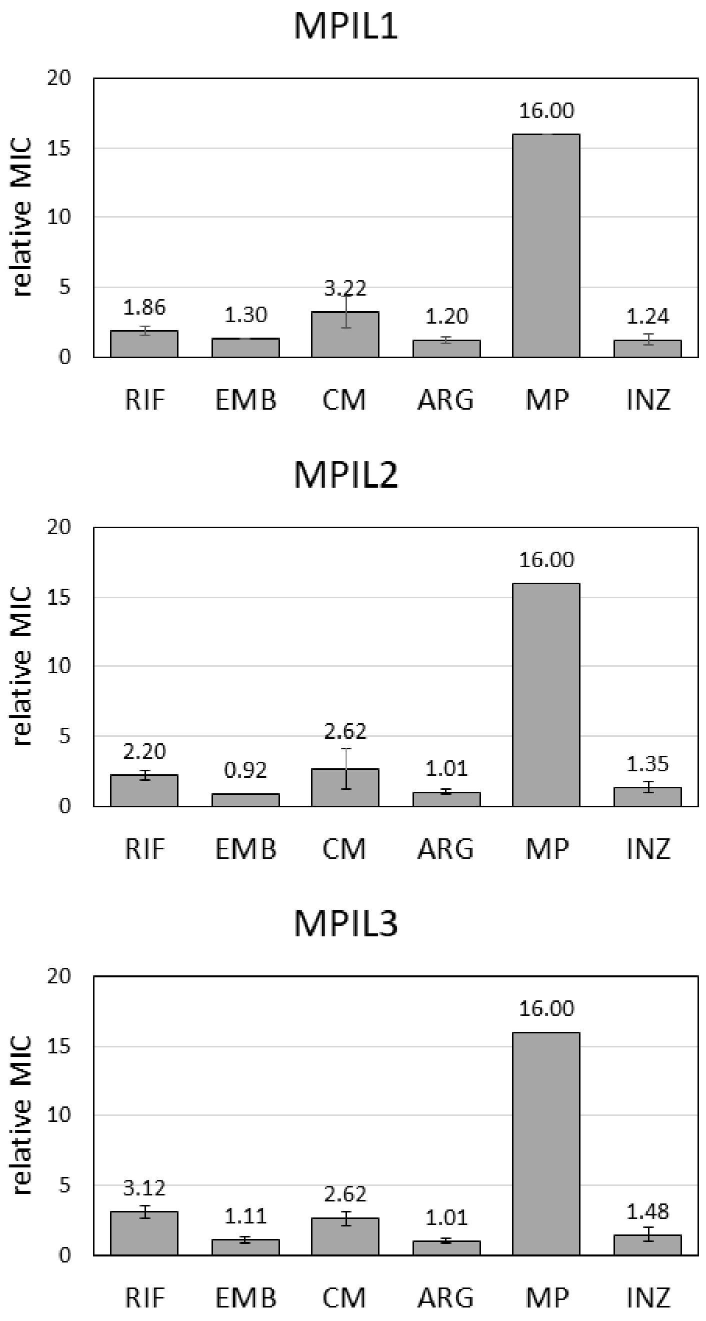
MPIL1
relative MIC
20
15
10
5
0
1.86
1.30
3.22
1.20
16.00
1.24
RIF
EMB
CM
ARG
MP
INZ
MPIL2
relative MIC
20
15
10
5
0
2.20
0.92
2.62
1.01
16.00
1.35
RIF
EMB
CM
ARG
MP
INZ
MPIL3
relative MIC
20
15
10
5
0
3.12
1.11
2.62
1.01
16.00
1.48
RIF
EMB
CM
ARG
MP
INZ

Fig S1. Early-stage of meropenem (MP) resistance acquisition in association with multidrug resistance.

The minimum inhibitory concentrations (MICs) of arginine (ARG), clarithromycin (CM), ethambutol (EMB), isoniazid (INZ), meropenem (MP), and rifampicin (RIF) for MP-resistant strains isolated from the MP-evolved populations at passage 2 (line 3) or 3 (line 1 and line 2) were determined. The relative MIC values (log_2 ratio) of the isolated clones (MPIL1 from line 1 at passage 3, MPIL2 from line 2 at passage 3, MPIL3 from line 3 at passage 2) compared to the wild-type strain are shown. The experiments were performed in triplicate.

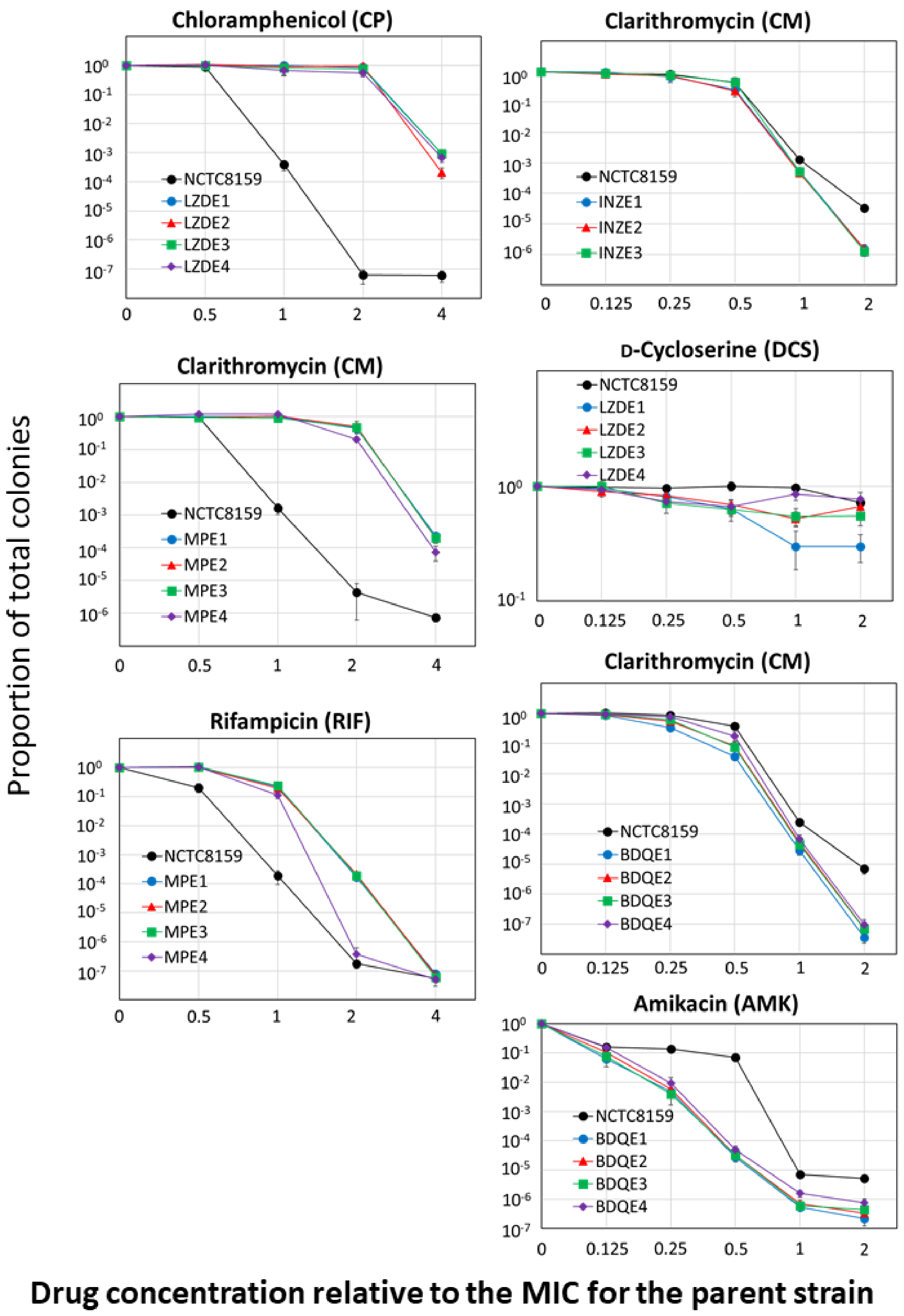
Chloramphenicol (CP)
NCTC8159
LZDE1
LZDE2
LZDE3
LZDE4
0 0.5 1 2 4
Clarithromycin (CM)
NCTC8159
INZE1
INZE2
INZE3
0 0.125 0.25 0.5 1 2
Clarithromycin (CM)
NCTC8159
MPE1
MPE2
MPE3
MPE4
0 0.5 1 2 4
D-Cycloserine (DCS)
NCTC8159
LZDE1
LZDE2
LZDE3
LZDE4
0 0.125 0.25 0.5 1 2
Rifampicin (RIF)
NCTC8159
MPE1
MPE2
MPE3
MPE4
0 0.5 1 2 4
Clarithromycin (CM)
NCTC8159
BDQE1
BDQE2
BDQE3
BDQE4
0 0.125 0.25 0.5 1 2
Amikacin (AMK)
NCTC8159
BDQE1
BDQE2
BDQE3
BDQE4
0 0.125 0.25 0.5 1 2
Proportion of total colonies
Drug concentration relative to the MIC for the parent strain

Fig S2. Validation of MIC determinations by measuring CFUs.

Colony-forming units (CFUs) of the parent strain (NCTC8159 strain) and the BDQ, INZ, LZD, and MP evolved strains for increasing concentrations of a respective drug. Diluted cells were dropped on R agar plates with increasing amounts of drugs (two-fold increments of MIC for the parent strain determined by measuring OD_{600} values). The CFU for each strain is normalized by the CFU of the no-drug condition. Data are presented as mean values +/- standard deviation for n=3 biologically independent samples.
